# Awareness and health risk protection behaviours of scavengers in the Gbalahi landfill site, Ghana, in the era of sustainable development

**DOI:** 10.1007/s43621-021-00070-7

**Published:** 2022-01-04

**Authors:** Joseph Gyea Nuripuoh, Abudu Ballu Duwiejuah, Noel Bakobie

**Affiliations:** 1 Department of Environment and Sustainability Sciences, Faculty of Natural Resources and Environment, University for Development Studies, Nyankpala Campus, Tamale, Ghana; 2 Department of Biotechnology, Faculty of Biosciences, University for Development Studies, Nyankpala Campus, Tamale, Ghana; 3 Department of Environment, Water and Waste Engineering, School of Engineering, University for Development Studies, Nyankpala Campus, Tamale, Ghana

**Keywords:** Health risks, Landfill, Perceptions, Solid waste, Waste scavengers

## Abstract

Waste picking is a pivotal in achieving sustainable waste management, environment health and economic development in the era of sustainable development. The study assessed the practices, knowledge, perception and health risk protection behaviours of waste scavengers in the Gbalahi landfill site. A total of 60 scavengers were conveniently sampled and interviewed. The study revealed that 93% of the waste scavengers sort waste using hooks and their bare hands. The study also showed 62% of the respondents have ever been physically abused by other scavengers. A good number of scavengers believed they have been fortified against “dirt diseases” during their childhood and have developed natural immunity against diseases. The knowledge of scavengers was skewed towards economic benefits as they viewed waste picking as a survival strategy. Discrimination and physical abuse posed a seemingly significant psychological health risk to majority of them. Safety and protection practices are limited to the use of pieces of clothes to cover the nose, wearing of multiple clothes and worn-out boots recovered from the landfill. Most of the respondents risk being exposed to the virus and pathogens. It is recommended that education and increased sensitisation should be encouraged and implemented by the Environmental Protection Agency (EPA), Ghana Health Service and other allied institutions in order to regularise and ensure the health and safety of waste scavengers.

## Introduction

Waste management as a priority is critical to addressing the Sustainable Development Goals (SDGs). Solid waste management is a major problem for most communities around the world and in recent years, world cities generate approximately 1.3 billion tonnes of solid waste every year, which is projected to increase to 2.2 billion tonnes by 2025 [1]. This observed phenomenon has significantly increased the cost of waste management globally. Globally, 70% of the total waste generated which is solid waste ends up at the landfill [1]. Most landfill sites in Ghana are essentially open dumps without leachate or gas recovery systems hence operate below the acceptable environmental health standards [2]. Waste picking therefore have the potential of reducing the quantum of waste in the environment, extending the lifespan of landfills, creating employment as well as generating economic returns [3]. Hence, the sustainable development goals can be accomplished when there is a high reduction of prevalence of devastating illnesses, and good population health that can be ensured through proper personal hygiene, good sanitation and safe environment.

Waste picking is a very important and common waste management activity in most developing countries and has a myriad of economic and environmental benefits. Waste picking provides employment for about 2% of the population in third world cities [4]. Cities such as Karachi, Manila, Jakarta and Bangkok save about $3 million dollars annually owing to the activities of scavengers [4]. The World Bank estimates that waste picking (waste recovery) provides a means of survival for about 1% of the population in African, Asian, and Latin American cities [5]. Recovered waste may be sold by scavengers for the purpose of recycling or reuse. This comes at the risk of adverse occupational and health hazards [3], however the activities of scavengers may seem to be sustainable waste management and development strategy.

Scavengers faced diverse problems and health hazards during waste picking which are threat to achieving some of the sustainable development goals such as access to decent work, clean water and sanitation, good health and well-being and reduced inequality. Six-point classification of hazards and risks to which scavengers can be exposed which include: occupational accidents (injuries such as cuts), physical risk caused by working under all sorts of weather, chemical risks (inhaling toxic gasses), psychological risks (sexual harassment, low self-esteem and hallucinations), biological risks (intestinal protozoa, helminthes, eye infections, skin diseases, diarrhoea and human immunodeficiency virus (HIV)/acquired immunodeficiency syndrome (AIDS)) and general hazards resulting from bites and stings from insects, scorpions, dogs, snakes amongst others [6].

Scavengers operate under perilous conditions without personal protective equipment (PPE) and are often prone to microbial infections, bites, cuts amongst others [5, 7]. Waste workers are exposed to different diseases which required improve health education and medical examination to help reduced it [8]. The solid waste scavenging have health implications and consequences on achieving the sustainable development goals. This study therefore sought to assess the practices, knowledge, perception and health risk protection behaviours of solid waste scavengers in the Gbalahi landfill site.

## Literature review

### Scavenging

A waste scavenger can generally be described as any person who is involved in the recovery or salvaging of materials with the potential for reuse and recycle in order to sell or for personal consumption [9]. Scavenging as an informal sector initiative in solid waste management, generally stems from the premise that some economically useful materials can be recovered from solid waste that has been discarded or disposed [10]. Scavenging has both economic and environmental benefits, it serves as a source of employment and income to unemployed individuals, supplying inexpensive raw materials to industries, it also reduces the demand for collection, transport and disposal equipment and facilities. The recycling of materials as a result of scavenging has a lower environmental impact as compared with the use of virgin resources. Scavenging is a common occurrence in third World countries owing to the prevalence of high unemployment, widespread poverty and the lack of a safety net for the poor [9, 11].

### Four motivational theories or schools of thought for waste picking

Navarrete-Hernandez [12] identifies four motivational theories or schools of thought for waste picking thus the dualist, structuralist, neoliberal and co-production theories. Dualist’s theorists argue that waste picking from the waste stream stems from stagnating economic growth and unavailability of formal employment. Dualist theorists further believe that, there is an inverse relationship between the number of people employed as waste pickers (scavengers) and economic growth. Dualist policies in relation to waste pickers are generally repressive and based on the premise that increased employment generation in the formal jobs would reduce the population of people employed as scavengers or waste pickers [12].

Structuralist’s perceive waste picking as major element of the capitalist system. Waste picking serves as a means of meeting the demand for recyclable materials from formal enterprises. Industries and other formal enterprises are able to reduce cost of production and increase returns owing to the availability of low-cost recyclable materials as a result of waste picking [12, 13]. Waste picking generally reduces the cost of production. Structuralist theorists are of the conception that, there exist a positive relationship between waste picking and economic growth. Structuralist policies promote waste-picker associations and unions, in order to reinforce waste pickers’ power to negotiate better prices and working conditions [12, 13].

Neoliberals perceive scavengers/waste pickers as small-scale entrepreneurs [14]. Based on this premise, scavenging/waste picking can be said to be intricately linked to the formal industry in the following ways. Firstly, waste picking at the industrial level provides local industries with affordable or cheap substitutes for raw materials thus reducing the cost of production and subsequently maximising profits and competitiveness at the industrial level. Secondly, the formal market of raw materials determines the types of substitute materials that are in demand and the prices paid to waste pickers and as such waste picking is inextricably linked to the level of competitiveness of local industries. Neoliberals posits that there is an inverse/negative relationship between waste picking and economic growth [14]. In times of economic crisis, the depreciation of local currencies results in an upsurge in the prices of imported raw materials and this in turn results in an increase in the demand for cheaper substitutes or raw materials recovered by scavengers/waste pickers. Neoliberals believe that waste picking is a highly efficient activity plagued by regulatory bottlenecks and a lack of legislation or policy direction resulting in the inability of waste pickers to achieve their utmost economic potential [12].

With co-production theory, a burgeoning number of researchers/academics are canvassing for the recognition and prioritisation of the role of the informal economy as an important player in the provision of public services in developing countries [12]. Joshi and Moore [15] argued that the monopoly enjoyed by the state in the provision of essential public services and the newfangled public management strategy of privatization have fallen short in providing standard public services in most developing countries as a result of logistical constraints and governance inefficiencies. Logistical constraints and failures maybe related to the provision of public services to deprived communities who are widely dispersed in terms of geography and do not have the capacity to pay for the services rendered. Governance inefficiency and failure on the other hand results from an institutional lack of capacity to ensure a sustained provision of public services whilst achieving a sustainable financing system [15].

The setbacks stems from the conventional “supply-led engineers” approach which is premised on capital intensive investments, high cost of operation and high standards for developing countries characterised by high availability of labour, low governance capacity and limited investment capacity [16]. Ostrom [17] posits that “co-production” arrangements through which a long-term partnership, citizens and the state are able to assemble resources to provide goods and services for the public, presents an intervening opportunity or solution for the delivery of basic services in developing countries. Joshi and Moore [15] proposed that co-production with the informal economy should not be overlooked since it has the prospects of being the best alternative in the provision of essential public services. Public sector support is pivotal in order to increase the productivity of waste-pickers under the co-production theory, consequently, this will optimise the economic efficiency, social equity and positive environmental impacts of waste picking.

### Health risks and hazards of waste picking

Health and safety is: “the promotion and maintenance of the highest degree of physical, mental and social well-being of workers in all occupations; the prevention amongst workers of departures from health caused by their working conditions; the protection of workers in their employment from risks resulting from factors adverse to health; the placing and maintenance of the worker in an occupational environment adapted to his physiological and psychological capabilities; and to summarise, the adaptation of work to man and of each man to his job” [9]. Health and safety generally involves the process of assessing risks and making changes to systems and mechanisms such that these risks are prevented or mitigated. Health risks maybe influenced by the following factors; the type of work, prevailing environmental conditions within which the work is situated, predisposed or pre-existing health conditions of the worker and resource availability to improve working conditions and maintain standards [9].

Health determines one’s mental and physical fitness and capability of functioning effectively for the good and benefit of society [18]. Health as [18] suggests, is dependent on the environment. Pandey [18] further suggests that, health is a product welfare. Lower welfare results in bad health and bad health limits the realisation of higher welfare. Health problems associated with solid waste picking were greatly influenced by the environment. A study conducted by the World Bank in a Mexico City dumpsite revealed that the average lifespan of waste pickers/scavengers on the landfill was about 39 years [19]. Binion and Gutberlet [20] classified the health risks and hazards associated with waste picking into six broad thematic areas which includes; chemical hazards, infection, ergonomic and musculoskeletal damage, mechanical trauma, emotional wellbeing and vulnerabilities, and environmental contamination.

## Methodology

### Study area

**Fig. 1:**
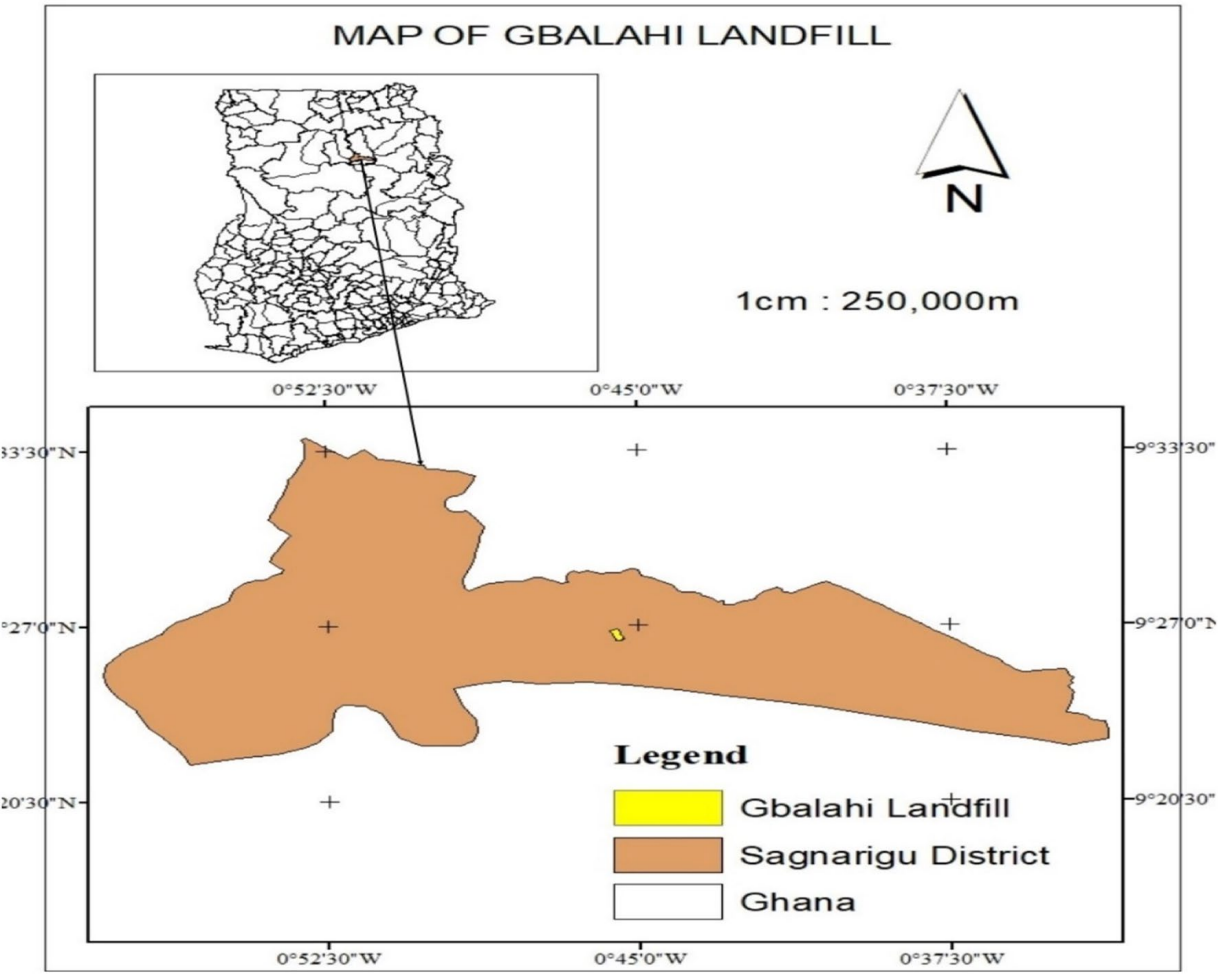
Map of the Gbalahi Landfill site

Tamale landfill site is located in Gbalahi in the Sagnarigu Municipality in the Northern region of Ghana. Gbalahi shares boundaries with Kulahi to the east, Taha to the west, Wuvogumani to the north and Mali to the South (Fig. 1) [21]. The landfill is located (Fig. 1) within latitude 9.441 and longitude − 0.759.

### Research setting, sample and data

The research was descriptive this was used to broaden the scope of understanding on the perception and health risks protection behaviours of waste picking in the Gbalahi landfill site. Qualitative methods are inductive since they seek to discover, not test, explanatory theories [22]. Primary data was collected from January to April, 2019 using preliminary reconnaissance field surveys, focus group discussions, questionnaires, face-to-face interviews and field observations. A preliminary reconnaissance survey was conducted to be appraised with the study area and resources needed to conduct the research. This was useful in the formulation of the questionnaire and interview schedules and in understanding and appreciating the scale of waste picking and the waste picking practices on the landfill site. It was also used to establish contact with the leadership of the scavengers and other key informants.

Convenience sampling method was used to sample scavengers on the landfill site. This sampling technique was used because there was a general sense of apprehension by the scavengers that the research and such studies were aimed at removing them from the landfill site or waste management stream. Furthermore, solid waste scavengers at the landfill were mostly preoccupied or busy sorting and sifting through waste on the landfill. It was therefore expedient to employ convenience sampling technique.

Permission was sorted and granted by the landfill managers to carry out the research. Scavengers consent were sorted and confidentiality assured before the face-face interview and focus group discussions. The questionnaire was developed, pre-tested and used to elicit relevant information from the solid waste scavengers. A total of 60 respondents were used owing to the fact that data and theoretical saturation had been reached. The saturation criterion is widely used in qualitative research to justify and validate sample size. Data and/or theoretical saturation is attained when in the process of data collection and/or analyses it suffixes that no new elements or additional information is been added or revealed. Thus, there appears to be information redundancy and further data collection becomes counterproductive and unethical [23]. Grady [24] agreed that data saturation is reached when “new data tend to be redundant of data already collected. In interviews, when the researcher begins to hear the same comments again and again, data saturation is being reached. It is then time to stop collecting information and to start analysing what has been collected”. To confirm saturation, data collection was continued for 15 more respondents.

The face-to-face interviews were conducted using an interview guide in order to have an in-depth awareness, knowledge, perception and modus operandi as related to waste picking on the landfill site. The focus group discussion was held with a cross section of the leadership of the scavengers on the landfill as well as other quasi women and youth groups (children) each made up of a maximum of 12 participants. This method was employed in order to explicate their concerns in terms of vulnerability, discrimination, emotional and physical abuse and how they think the adverse health impacts of waste picking may be augmented. Periodic visits were made to the landfill site in order to understand and better appreciate the scope of waste picking activities on the landfill site. This was done alongside with keen observation of the use of personal protective equipment, scavenger-scavenger interactions or relationship, how waste was sorted and processed on-site amongst others.

### Measures of variables

The questionnaire contained both open and close ended questions and categorised into four main thematic areas thus; Part A: Demographic characteristics (gender, age, marital status and education levels of scavengers), Part B: Socio-economic status (occupation, income, working days, duration and period, marital status and education levels of scavengers), Part C: Attitudes, knowledge, practices, perception and motivation, and Part D: Health risk, protection behaviours and safety practices.

### Data analysis procedure

The statistical package for social sciences (SPSS version 25) and Microsoft office excel 2019 were used to process the quantitative data into graphs, charts, and tables for interpretation and discussion. Correlation analysis was also conducted to identify any significant statistical relationship between the study variables.

## Results and discussion

### Socio-demographic and economic characteristics of scavengers

The study revealed that 63% of the respondents were males and 37% were females (Table 1). The study showed scavengers within the age range of less than 15 years were about 37%, 16–25 years were 20%, 26–35 and 36–45 years each representing about 22% (Table 1). Majority of the respondents were males and all the respondents were in their youthful age. This finding agrees with that of [7, 25] who attributed the male dominance and youthfulness to the fact that waste picking is labour intensive which requires a lot of physical strength. It may be due to the unemployment rate in Ghana and as such the youth are compelled to engage in waste picking so as to make ends meet.

**Table 1:** Socio-demographic characteristics of scavengers in the Gbalahi Landfill Site

	Frequency (N = 60)	Percent (%)
Gender		
Male	38	63.30
Female	22	36.70
Total	60	100
Age		
Less than 15 years	22	36.70
16–25 years	12	20.00
26–35 years	13	21.70
36–45 years	13	21.70
Total	60	100
Level of education		
No formal education	23	38.30
Primary	21	35.00
JHS/SHS	16	26.70
Total	60	100
Marital status		
Married	23	38.30
Single	36	60.00
Divorced	1	1.70
Total	60	100

The study observed 37% of the scavengers which is the highest age range were less than 15 years. This finding contrast with that of [25, 26] that observed that majority of scavengers were between the ages of 20–30 years, 31–40 years and 20–29 years, respectively. Majority of the respondents between the ages of 5–25 years had formal education of which 57% was primary education (Table 1). Some of the respondents with formal education were dropouts due to lack of financial support and interest in formal education. This finding is similar to that of [7, 10, 25, 27, 28] that observed a high percentage in educated scavengers thus 98%, 87%, 81%, 87% and 71% with a significant proportion been at the primary school level. This finding however contradicts that of [29] who observed that about 60% of the scavengers in Obio/Akpor local government in Nigeria did not have any formal education. Correlation analysis showed a strong negative correlation between the age of the respondents and their level of education (r = − 0.661**). The number of single, married and divorced scavengers accounted for 60%, 39% and 1% of the total respondents, respectively (Table 1).

Most of the respondents were students and few were into farming, trading, waste picking, and artisanship as their main occupation (Table 2). Almost all the respondents alluded to the fact that besides their main occupation they were also into farming. This was corroborated by the reduced waste picking activity during the rainy season since most of them had to attend to their farms.

**Table 2:** Socio-economic characteristics of scavengers in the Gbalahi Landfill Site

	Frequency (N = 60)	Percent (%)
Main occupation		
Farming	17	28.30
Trading	10	16.70
Waste picking	9	15.00
Student	22	36.70
Artisanship	2	3.30
Total	60	100
Average monthly income of respondent (GH¢1 = 0.16 USD)		
GH¢ 1–100 (0.16–16.35 USD)	50	83.30
GH ¢ 101–300 (16.52–49.06 USD)	10	16.70
Total	60	100
Working days/week		
Once/week	7	11.70
2–3 days/week	27	45.00
4–6 days/week	20	33.30
7 days/week	6	10.00
Total	60	100
Working hours/day		
4–6 h/day	37	61.70
7–12 h/day	23	38.30
Total	60	100
How long have you been in this occupation		
Less than 12 months	8	13.30
1–3 years	22	36.70
4–6 years	10	16.70
7–10 years	10	16.70
10 years and above	10	16.70
Total	60	100

The respondents that had an average monthly income between GH¢ 1.00 to 100.00 (0.16–16.35 USD) were 83% and only few (17%) of them earned a monthly average between GH¢ 101.00 to 300.00 (16.52–49.06 USD) (Table 2). The average monthly income levels of scavengers may be an influential factor in the burgeoning increase in the number of scavengers. This has the potential to increase if waste picking on the landfill is made less perilous and more efficient through the provision of waste picking tools and protective equipment.

The study showed that about 99% of the respondents lived in close proximity to the landfill and were natives with 95% living in their family house and the remainder 5% living in their own house/residence. The study further revealed that, 45% of the respondents work 2—3 days per week, 33% of scavengers work 4—6 days per week with 12% and 10% of the respondents working once a week and 7 days a week, respectively (Table 2). The respondents that work 4—6 h per day were 62% and 38% of the respondents worked 7–12 h per day. The study revealed that some of the respondents within school going age were on the landfill waste picking for waste during school days. Hence, without proper regulatory measures, waste picking maybe an incentive for children of school going-age to be truant in schools and possibly drop-out of school. Reports from the Ghana Education Service (GES) and Education Management Information Systems (EMIS) data of the world bank shows that, the drop-out rate in Ghana from primary 1 to 4 remains fairly stable (1%) but rises from 3% in primary 5 to 12% in primary 6. In the Junior High Schools, the drop-out rate increases significantly from 3% in JHS 1 to 23% in JHS 3 [30–32].

The study also showed that about 50% of the respondents have been working as scavengers on the landfill for a period not less than four years, 37% have been working as scavengers for 1—3 years and the remainder 13% working for less than a year (Table 2). Half of the respondents working as scavengers on the landfill for not less than four years. This finding is similar to researches conducted by [25, 27]. This may have dire implications since long term exposure to landfill gases and other biohazards is detrimental to their health. The respondents affirmed there is readily available market for recovered waste materials (Table 2).

### Practices, knowledge, perceptions and motivation

Almost all the respondents scavenge waste using hooks and their bare hands (Table 3). Waste is sorted out using sticks, hooks and sometimes with the bare hands and with relatively no protective gear. The respondents also alluded that sometimes they deliberately set fire to heaps of waste on the landfill to make easy to spot metals. This practice could pose a great health and environmental risk to the scavengers and the general public [5].

**Table 3:** Practices, knowledge and perception of scavengers in the Gbalahi Landfill Site

	Frequency (N = 60)	Percent (%)
How do you sort or sift through waste		
Hooks	4	7.00
Hooks and bare hands	56	93.00
Total	60	100
What materials do you often recover from the landfill		
Plastics	36	60.00
Metals	24	40.00
Total	60	100
Do you use personal protective equipment during waste picking		
Yes	23	38.30
No	37	61.70
Total	60	100
If yes how often		
Always	13	57.00
Sometimes	10	43.00
Total	23	100
Do you know the importance of personal protective equipment		
Yes	58	96.70
No	2	3.30
Total	60	100
Do you know that landfills are a source of pathogens and vectors		
Yes	58	96.70
No	2	3.30
Total	60	100
Do you know that hazardous waste is disposed at the landfill		
Yes	57	95.00
No	3	5.00
Do you know that long term exposure to landfill gas is detrimental to your health		
Yes	48	80.00
No	12	20.00
Total	60	100

Most of the respondents often recover plastics whilst few recover metals (Table 3). This finding corroborates that of [27] who found 25% of the respondents often picked plastics and 12.50% of them picked scrap metals and iron ore. Contrarily, [26] reported that metals were the most recovered material followed by plastics and bottles. Waste pickers/scavengers recycle about 50% of plastics in developing countries, which is about five times greater than the plastic recycling rate in the United States [33]. This study revealed a good number of recovered plastics were pesticides and herbicide containers. This observation highlights a significant threat of exposure to toxic and possible carcinogenic organic compounds contained in these containers.

Majority of the respondents do not use any personal protective equipment (Table 3) with most of them less than 15 years of age. This finding learns support from [5, 25] that reported that scavengers do not use personal protective equipment. This study also revealed that the respondents do so because of the believed that they have been fortified (using herbs) against “dirt diseases” during their childhood and they have developed natural immunity against these diseases. Marello and Helwege [34] attributed this phenomenon to physical debilitation, lack of education to accurately assess risks, emotional disabilities and income imperatives, which prevent scavengers from protecting themselves. Children owing to their size, physiology and behaviour are more susceptible and vulnerable to environmental hazards [35]. This therefore brings into sharp focus their vulnerability to a myriad of health risks and occupational hazards because of their young age.

Majority of the respondents mentioned income and employment as the importance of waste picking (Table 4). Only few of them said it helps reduce waste on the landfill site. The respondents suggested source of income to be their motivation for waste picking. Similar study by [10] observed 31.50% of the respondents in a Lagos dumpsite said their reason or motivation for waste picking was economic (based on the need to survive).

**Table 4:** Motivation for waste picking in the Gbalahi Landfill Site

	Frequency (N = 60)	Percent (%)
The importance of waste picking		
Source of income and employment	58	98.00
Reduces waste on the landfill	2	2.00
Total	60	100
Are you happy with this work		
Yes	17	28.30
No	43	71.70
Total	60	100
Do you wish to leave this occupation when presented with an alternative		
Yes	57	95.00
No	3	5.00
Total	60	100

Most of the respondents said they were not happy working as scavengers. This was supported by the fact that almost all the respondents expressed interest in leaving the occupation when presented with an alternative (Table 4). Even though waste picking may be beneficial economically and environmentally, a good number of scavengers are not happy with their current working conditions and find waste picking unpleasant. This finding contradicts that of [27] who reported that about 90% of the respondents said they found waste picking pleasant.

### Health risk, protection behaviour and safety practices

All the respondents practice hand washing with soap (Table 5). This response does not reflect the reality on the ground owing to the unavailability of clean potable water around the landfill site. Almost all of the respondents always take their bath with few taking their bath sometimes (Table 5). Some respondents however alluded that other members of their households will not allow them to touch materials in the house till they washed their hands or took their baths. Majority of the respondents do not wash their clothes at all since those clothes are only wore to the landfill for waste picking. This practice can be a source of pathogens that cause skin diseases.

**Table 5:** Health risk, protection behaviour and safety practices in the Gbalahi Landfill Site

	Frequency (N = 60)	Percent (%)
Do you eat or cook on the landfill		
Yes	53	88.30
No	7	11.70
Total	60	100
How often do you wash your clothes		
Sometimes	25	41.70
Not at all	35	58.30
Total	60	100
How often do you take your bath		
Always	57	95.00
Sometimes	3	5.00
Total	60	100
Have you been physically abused by others in your field		
Yes	37	61.70
No	23	38.30
Total	60	100
Are you discriminated against because of waste picking		
Yes	25	41.70
No	35	58.30
Total	60	100
Are you emotionally/psychologically affected by waste picking		
Yes	20	33.30
No	40	66.70
Total	60	100
Do you use drugs or medicines in order to help you work		
Yes	6	10.00
No	54	90.00
Total	60	100
Are you adversely affected by the weather		
Yes	58	96.70
No	2	3.30
Total	60	100
Have you been pierced by a hypodermic object (syringe) or a sharp object		
Yes	48	80.00
No	12	20.00
Total	60	100
Have you been bitten by an animal or insect during waste picking		
Yes	49	81.70
No	11	18.30
Total	60	100
Have you accidentally fallen whilst waste picking on the landfill		
Yes	39	65.00
No	21	35.00
Total	60	100
Have you accidentally ingested any liquid or chemical whilst waste picking		
Yes	18	30.00
No	42	70.00
Total	60	100
Averagely how much do you spend on medication monthly (GH¢)		
1–50	56	93.30
51–100	4	6.70
Total	60	100
Do you have health insurance		
Yes	36	60.00
No	24	40.00
Total	60	100

**Fig. 2:**
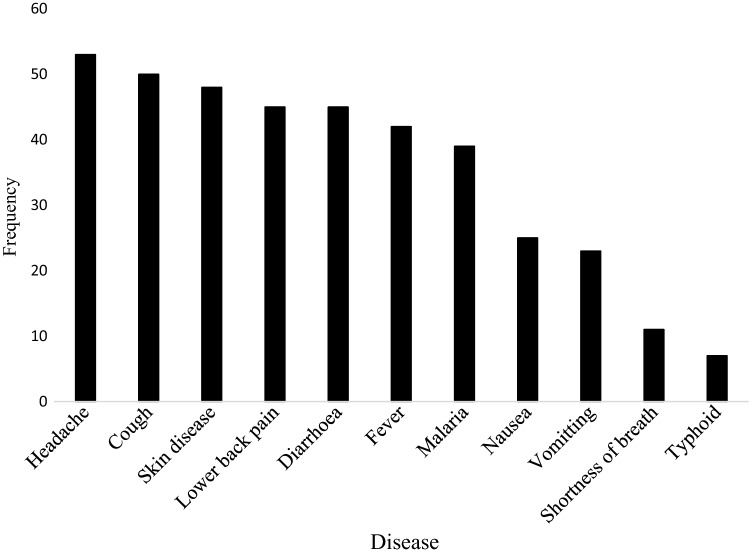
Diseases/health conditions recorded by the scavengers in the last 12 months in the Gbalahi Landfill Site

Most of the respondents eat or cook in the landfill site, this was mostly practice by nursing mothers and children. This practice poses a health threat since flies were mostly hovering around and could transmit other diseases. This is could be the reason why most of them complained of diarrhoea (Fig. 2).

Most of the respondents have experienced physical abuse by other scavengers (Table 5). This sometimes results from misunderstandings about ownership of recovered waste materials and bullying. Almost half of the respondents have been discriminated against because of waste picking with a significant. The study showed a weak but significant negative correlation between age of respondents and psychological/emotional trauma (r = − 0.282*). Thus, the younger population are most likely to be emotionally or psychologically affected by waste picking. According to the [36], depression is one of the leading causes of illness and disability among adolescents, and suicide is the second leading cause of death in adolescents. Violence, poverty, humiliation and feeling devalued can increase the risk of developing mental health problems amongst children and adolescents [36]. This observed phenomenon may go a long way in negatively impacting their mental health and education. However, majority of the respondents do not seek counseling. This finding corroborates that of [37] who posit that children and adolescent mental health (CAMH) disorders are becoming prevalent globally. However, access to mental and psychological health care especially in developing countries like Ghana is generally limited and inadequate. The treatment gap for mental and psychiatry related disorders is at about 98% [38].

Majority of the respondents do not use drugs such as antidepressants, painkillers, alcohol, tobacco amongst others to help them in waste picking. However, few of them sometimes use painkillers which have not been prescribed by a professional physician. This was because of the tedious nature of waste picking and as a result they usually end up with general body pains. The observed practice presents a threat of misdiagnoses and drug abuse which might have adverse health impacts on their health in the long run.

Almost all the respondents complained about the weather which they said has dire impacts on their health (Table 5). Majority of the respondents alluded to the fact that they have been pierced by a hypodermic or sharp object during waste picking (Table 5). This poses a health threat since hospital waste are disposed of in the landfill. Scavengers can be exposed to hazardous waste materials in the landfill which may be a transmission point of diseases such as the dreaded acquired immunodeficiency syndrome (HIV/AIDS), tetanus, amongst others. Since, [39] reported that some health facilities do no properly segregate and/or incinerate waste before disposal in the landfill site. Scavengers are usually victims of bee stings and mosquito bites. This may be the reason why scavengers complained of malaria cases. There have been a few instances of snake and scorpion bites on the landfill and the absence of a first aid box makes these occurrences potentially fatal.

Majority of the scavengers have fallen whilst waste picking (Table 5), as they tried to ascend and/or descend the heap of waste or trying to lift heavier objects than they can carry. Few of the respondents have accidentally ingested chemicals whilst waste picking that have accidentally splashing into their mouths. All the respondents alluded that they are irritated by the obnoxious odour from the landfill.

Majority of the respondents have health insurance and spend between GH¢ 1.00 to GH¢ 50.00 a month on health (Table 5). This is about half of their monthly income and expenditure on health could increase in the long run if safety and health precautions are not strictly adhered to. Some diseases or health conditions experienced by the respondents within the last 12 months (Fig. 2).

### Covid-19 risk behaviour and waste generation

The Covid-19 pandemic has disrupted and negatively affect a myriad of human livelihood including issues bothering on waste management. The increased production and use of PPEs as well as the changes in consumption patterns in major cities as a result of lockdown measures imposed by authorities has significantly impacted the rate and composition of waste generation globally. It is reported that, 129 billion face masks and 65 billion gloves are used every month worldwide [40]. The Asian Development Bank (ADB) reported that there has been a marked increase in waste and biomedical wastes in most Asian cities. In the Hubei province for instance infectious medical waste increased by about 6 folds as a result of Covid-19. This observed waste generation pattern has varied implications as far as sustainable waste management and waste picking is concerned [41, 42].

It was observed that in terms of Covid-19 risk behaviours, majority of the respondents risk being exposed to the virus and pathogens. As, the disposal of biomedical waste at the landfill has been reported [39]. Improper disposal of healthcare and biomedical wastes is strongly associated with public health outcomes [43, 44]. Similarly, disposable facemasks, face-shields, used tissues and reusable facemasks are becoming a major part of domestic household waste and may serve as contact sources or points of the covid-19 virus. The UNEP [41] reports that there is an increase in the amount of mixed waste (infectious wastes inclusive) owing to reduced waste segregation at source. The tendency to reuse scavenged or salvaged waste materials especially reusable facemasks and face shields as observed is a risk factor in terms of exposure of the waste scavengers to Covid-19. The rush to salvage waste whenever skip trucks freshly dispose of waste and the grouping of scavengers under trees and makeshift structures without recourse to social/physical distancing protocols is a major risk factor observed. The inadequate use of PPEs and poor handwashing practices owing to the absence of potable water and handwashing facilities on site pose risk for the scavengers. The length of time spent at the landfill is also a major risk factor. The perception that they have been fortified against “dirt” diseases through the use of herbs during childhood and the lack of awareness about the effects and risks of Covid-19 maybe reasons accounting for the observed indifference towards ensuring their own personal safety and protection. Rahman et al. [45] posits that informal waste workers are at a high risk of been exposed to Covid-19 (SARS-CoV-2) owing to the nature of their work and prevailing working conditions.

## Conclusion

Scavengers in the landfill play a very significant and pivotal role in sustainable waste management and ensuring environmental health. Scavengers have fair knowledge about the health hazards associated with waste picking but believed it has an insignificant threat to their physical, social and mental well-being. Scavengers are motivated by the monetary or economic gains. A good number of scavengers on the landfill do not use personal protective equipment. Safety and protection behaviour and practices are limited to the use of pieces of clothes to cover the nose, wearing of multiple clothes and worn-out boots recovered from the landfill. Hand washing culture is often practiced by scavengers off-site but sparingly not in the landfill site. Occupational health hazards associated with waste picking are biological, physical and psychological. Scavenging is grossly unsanitary, unhygienic, and scavengers gets low income from waste resources recovered, they suffer physically and psychological stigma and also lack protective gears. These threatened the sustainable development goals which hitch decent work, clean water and sanitation, good health and well-being and reduced inequality. Covid-19 health risks behaviours, majority of the respondents risk being exposed to the virus and pathogens. It is recommended that education and increased sensitisation should be encouraged and implemented by the Environmental Protection Agency (EPA), Ghana Health Service and other allied institutions in order to regularise and ensure the health and safety of waste scavengers. Scavengers less than 15 years old should not be allowed to scavenge in accordance to Sects. 58 to 61 of the labour Act.

## Data Availability

If requested, we are available to disseminate the data of the paper.
